# Health and economic outcomes of treatment with extended-release naltrexone among pre-release prisoners with opioid use disorder (HOPPER): protocol for an evaluation of two randomized effectiveness trials

**DOI:** 10.1186/s13722-020-00188-5

**Published:** 2020-04-22

**Authors:** Sean M. Murphy, Philip J. Jeng, Sabrina A. Poole, Ali Jalali, Frank J. Vocci, Michael S. Gordon, George E. Woody, Daniel Polsky

**Affiliations:** 1 Department of Population Health Sciences, Weill Cornell Medical College, 425 East 61st Street, Suite 301, New York, NY 10065 USA; 2 Department of Psychiatry, Perelman School of Medicine, University of Pennsylvania, Philadelphia, PA USA; 3 Friends Research Institute, Baltimore, MD USA; 4 Department of Health Policy and Management, Bloomberg School of Public Health, Carey Business School, Johns Hopkins University, Baltimore, MD USA

**Keywords:** Opioid use disorder, Justice involved persons, Extended-release naltrexone, Healthcare utilization, Health-related quality-of-life, Cost-effectiveness

## Abstract

**Background:**

Persons with an opioid use disorder (OUD) who were incarcerated face many challenges to remaining abstinent; concomitantly, opioid-overdose is the leading cause of death among this population, with the initial weeks following release proving especially fatal. Extended-release naltrexone (XR-NTX) is the most widely-accepted, evidence-based OUD pharmacotherapy in criminal justice settings, and ensures approximately 30 days of protection from opioid overdose. The high cost of XR-NTX serves as a barrier to uptake by many prison/jail systems; however, the cost of the medication should not be viewed in isolation. Prison/jail healthcare budgets are ultimately determined by policymakers, and the benefits/cost-offsets associated with effective OUD treatment will directly and indirectly affect their overall budgets, and society as a whole.

**Methods:**

This protocol describes a study funded by the National Institute of Drug Abuse (NIDA) to: evaluate changes in healthcare utilization, health-related quality-of-life, and other resources associated with different strategies of XR-NTX delivery to persons with OUD being released from incarceration; and estimate the relative “value” of each strategy. Data from two ongoing, publicly-funded, randomized-controlled trials will be used to evaluate these questions. In Study A, (XR-NTX Before vs. After Reentry), participants are randomized to receive their first XR-NTX dose before release, or at a nearby program post-release. In Study B, (enhanced XR-NTX vs. XR-NTX), both arms receive XR-NTX prior to release; the enhanced arm receives mobile medical (place of residence) XR-NTX treatment post-release, and the XR-NTX arm receives referral to a community treatment program post-release. The economic data collection instruments required to evaluate outcomes of interest were incorporated into both studies from baseline. Moreover, because the same instruments are being used in both trials on comparable populations, we have the opportunity to not only assess differences in outcomes between study arms within each trial, but also to merge the data sets and test for differences across trials.

**Discussion:**

Initiating XR-NTX for OUD prior to release from incarceration may improve patient health and well-being, while also producing downstream cost-offsets. This study offers the unique opportunity to assess the effectiveness and cost-effectiveness of multiple strategies, according to different stakeholder perspectives.

## Background

The US is in the middle of an opioid epidemic, with an estimated 2.1 million US persons (aged 12 years and older) experiencing opioid use disorder in 2017 [1], and 47,600 people dying from a drug overdose attributed to opioids, which represents an increase in the age-adjusted rate of almost 400% since 2000 [2]. Opioid misuse has been associated with violent behavior and increased criminal activity [3–7]; concomitantly, approximately 50% of inmates suffer from a drug use disorder, versus only 2% of the general U.S. population [8]. Individuals being released from incarceration face many unique challenges to remaining abstinent from opioids [9–11]. For example, persons who were incarcerated are faced with stigma, they must secure housing and employment, and they must abide by other supervision requirements associated with their release, all while trying to re-affiliate themselves with family and friends. These challenges, combined with the fact that, while incarcerated, persons with a history of opioid use disorder typically do not lose their opioid cravings, but may lose their tolerance, result in high rates of opioid relapse, overdose, and overdose death following release from incarceration [12–14]. In fact, opioid-overdose is the leading cause of death among persons who were incarcerated, with the first 2 weeks following release from incarceration proving especially fatal [14–16].

Methadone, buprenorphine, or naltrexone pharmacotherapy is recommended as the first-line treatment for opioid use disorder [17]. Methadone and buprenorphine are effective, and cost-effective pharmacotherapies for opioid use disorder [7], and have been shown to be associated with increased rates of entry to community-based treatment [13, 18, 19], treatment retention [20–22], and opioid abstinence [13, 20, 21], when provided to persons who are incarcerated, just prior-to or immediately-following release; however, these medications are often unavailable to justice-involved populations [23, 24]. According to a 2003 nationwide survey, only 1% of persons who were incarcerated and reported regular opioid use received methadone or buprenorphine treatment [23, 25]. A more recent survey of a convenience sample of 18 jails and 12 prisons associated with an ongoing research study, revealed that one-third of the facilities were offering opioid use disorder pharmacotherapy to persons who entered incarceration on medication, and only one offered pharmacotherapy to persons who did not enter on a medication [26]. Two of the most commonly cited reasons for this lack of support for opioid agonist therapy were a preference for drug-free detoxification, and security concerns about providing the medications within the jail or prison [23, 26]. The security concerns likely stem from the fact that buprenorphine and methadone are narcotics, and therefore the incentive to misuse or divert the medications, exists. Furthermore, both methadone and buprenorphine have added legal barriers. Methadone can only be prescribed through a certified opioid treatment program, while buprenorphine requires the provider to obtain a waiver under the Drug Addiction Treatment Act of 2000 [27]. Moreover, the majority of institutions do not even refer individuals to clinics/providers offering pharmacotherapy, upon release [23], and, among justice-involved persons who are referred to specialty treatment for opioid use disorder, findings suggest that less than 5% receive methadone or buprenorphine, compared to 41% of those referred from other sources [28].

Naltrexone has some advantages as an opioid use disorder pharmacotherapy that make it more attractive to many prison/jail systems; it is a full opioid antagonist and therefore is non-narcotic and non-addictive [29], it is relatively non-stigmatized [30], and it can be prescribed by any healthcare provider licensed to prescribe medications [29]. Additionally, the extended-release naltrexone (XR-NTX) injection provides persons being released from incarceration with approximately 30 days of protection from opioid overdose. However, the high price of XR-NTX may serve as barrier to access. The upper-bound, wholesale acquisition cost of one XR-NTX injection is $1309 [31], while the lower-end cost to the Department of Veterans Affairs is $897 [32]. To put the price of XR-NTX in context, at the time of the aforementioned national survey of prison systems [23], the wholesale acquisition cost of name-brand buprenorphine-naloxone (Suboxone^®^) was only $200 per month [33], and 18% of prison systems indicated the cost of buprenorphine was prohibitive.

It is critical that the policymakers who are ultimately setting prison/jail healthcare budgets [34], and making decisions on behalf of taxpayers and society as a whole, do not view the cost of opioid use disorder therapy for the high-risk population of persons being released from incarceration, in isolation. This protocol describes a study funded by the National Institute of Drug Abuse (NIDA), which will use data from two ongoing randomized-controlled trials assessing different models of XR-NTX treatment among persons being released from incarceration, to evaluate the following questions: (a) what are the costs to the correctional health system of implementing and running each XR-NTX program; (b) do the programs produce downstream savings for state governments from reduced utilization of high-cost healthcare services, such as emergency department (ED) visits and inpatient stays [7], criminal activity [3–7], and recidivism [7, 35, 36]; (c) do the programs produce additional benefits for participants and society, such as enhanced quality of life, reduced risk of overdose/overdose death [3, 5, 7, 14], and improved workplace or school productivity [7]; and (d) under what circumstances are the programs cost-effective from the perspectives of state policymakers, and society.

## Methods/Design

### Overview

The economic analyses will follow recommendations of the Second Panel on Cost Effectiveness in Health and Medicine [37], Glick et al. [38], and Drummond [39], Data from two ongoing, publicly-funded, randomized controlled effectiveness trials in which XR-NTX is being evaluated among persons being released from incarceration who have an opioid use disorder, will be used to achieve the study objectives. In Study A, titled “Extended Release Injectable Naltrexone Before vs. After Release: A Randomized Trial of Opioid Addicted Prisoners” (Woody, PI; PCORI-1409-21688; XR-NTX Before vs. After Reentry), those randomized to the pre-reentry XR-NTX arm get their first dose prior to reentry and a referral to post-reentry treatment; the post-reentry XR-NTX arm is referred to a local provider to receive their first dose following reentry. In Study B, titled “Long-Acting Naltrexone for Pre-Release Prisoners: A Randomized Trial of Mobile Treatment” (Gordon, PI; R01DA040636; enhanced XR-NTX vs. XR-NTX), both arms receive XR-NTX prior to reentry; following reentry, the enhanced arm receives monthly mobile medical XR-NTX treatment at the participant’s place of residence, while the XR-NTX arm receives referral to a community opioid treatment program. Specifically, we will evaluate whether enhanced XR-NTX with mobile medical treatment for opioid use disorder among persons being released from incarceration is associated with more primary and behavioral healthcare services, but fewer emergency and inpatient services; enhanced participant wellbeing; and economic viability from state policymaker and societal perspectives, compared to (a) XR-NTX prior to reentry plus referral to post-reentry treatment, and (b) referral to post-reentry treatment only. We will also calculate the costs of implementing and continuously managing each pre-release XR-NTX intervention from the perspective of the correctional health system.

We chose to focus on the state policymaker and societal perspectives, as opposed to the healthcare sector perspective for example, because they are particularly relevant for opioid use disorder interventions in this population. However, given the information we are collecting, it will be possible to examine value from other perspectives, if necessary. The state policymaker perspective is crucial to informing resource allocation decisions on behalf of the public, who is primarily responsible for funding prison/jail interventions for substance use disorders, subsequent therapy, and other healthcare for formerly incarcerated persons, as well as the direct costs associated with recidivism. In our prior study of community-dwelling, justice-involved persons with an opioid use disorder, 98% were either on Medicaid or uninsured [40, 41]. The state policymaker perspective will include all study and non-study healthcare costs (e.g., opioid and other substance use disorder therapy; inpatient, outpatient, and ED services; behavioral therapy) paid by Medicaid, and all direct costs to the criminal justice system. In addition to the costs included in the state policymaker perspective, the societal perspective accounts for many of the indirect costs associated with opioid misuse, regardless of who shares the burden [37]; thus, it will also include indirect costs associated with criminal activity (e.g., property damage, pain and suffering, etc.), reduced workplace and school productivity, and those associated with participant time and travel to receive treatment. Not accounting for these indirect costs and the potential value offsets can undermine the true benefit of an intervention. Participant-level costs will be estimated using the resource costing method, which entails weighting each resource unit consumed by a pre-determined unit cost, and summing the values over the relevant time period [37, 38, 42].

### Overview of study A (Woody, PI): XR-NTX before vs. after reentry

This clinical trial is enrolling 200 persons being released from incarceration who met study admission criteria and expressed interest in XR-NTX treatment. Participants were stratified by sex and sentence requirements for post-reentry criminal justice system contacts (i.e. whether or not required to meet with probation/parole officer) and randomized 1:1 to (a) XR-NTX prior to reentry plus referral to post-reentry treatment, and (b) referral to post-reentry treatment only. Those assigned to the pre-reentry XR-NTX arm receive XR-NTX before reentry with the offer of 3 additional doses of XR-NTX delivered at a community opioid treatment program after reentry. Those assigned to the post-reentry arm are given an appointment to be seen at a community opioid treatment program where they can be admitted within 1–3 days of reentry to receive their first dose of XR-NTX, with the offer of 3 additional doses at the community treatment center. The primary outcome is relapse during the first 3 months after reentry.

### Overview of study B (Gordon, PI): enhanced XR-NTX with mobile medical treatment vs. XR-NTX

This clinical trial is enrolling 180 prisoners who meet study admission criteria and express interest in XR-NTX treatment. Participants are being stratified by sex and randomized 1:1 to enhanced XR-NTX or XR-NTX. Those assigned to enhanced XR-NTX receive one injection of XR-NTX in prison followed by 6 monthly injections post-release at the participant’s place of residence via mobile medical treatment. Those assigned to XR-NTX receive one injection of XR-NTX in prison, followed by 6 monthly injections post-release at a community opioid treatment program. The primary outcomes being evaluated are XR-NTX treatment adherence (number of injections received), opioid abstinence, criminal activity (self-report days), re-arrest and re-incarceration (official records), and HIV risk-behaviors (self-report).

### Inclusion/exclusion criteria for studies A and B

The inclusion criteria for Studies A and B include: adult males or females who are incarcerated, and eligible for release within 30–60 days; history of DSM-V opioid use disorder; interested in, and suitable for XR-NTX treatment as determined by medical evaluation; detoxified and able to pass a naloxone challenge; able to provide informed consent; willingness to enroll in XR-NTX treatment in prison [not currently in or planning to pursue agonist (methadone, buprenorphine) treatment at release]; and planning to live in the study area during the study period. Potential participants who meet any of the following will be excluded: renal disorder; active medical illness that may make participation hazardous (e.g., unstable diabetes, heart disease); untreated psychiatric disorder that may make participation hazardous (e.g., untreated psychosis, bipolar disorder with mania); history of allergic reaction to XR-NTX; current chronic pain diagnosis for which opioids are prescribed; creatinine above normal limits; pregnancy (for women); breast-feeding (for women); suicidal ideation (within the past 6-months); Body Mass Index (BMI) > 40; or unadjudicated charges that may result in transfer to another facility or additional prison time.

## Measures

The Participant Data Collection Schedule (Table 1) contains a list of measures relevant to this project and when they will be collected from participants for each study. Additionally, we will administer the Drug Abuse Treatment Cost Analysis Program (DATCAP) instrument at the program level for each trial, once the programs have reached a steady state. Self-report instruments of healthcare resource utilization and criminal activity will utilize recall periods anchored at the last assessment, in order to capture information that would be missed by standard 30-day recall periods, in the event a participant fails to attend a study visit.

**Table 1:** Data Collection Schedule

	Month															
	0		1		2		3		4		5		6		7	12
	Study															
	A	B	A	B	A	B	A	B	A	B	A	B	A	B	B	B
Non-study Medical and Other Services form (self-report)	**√**	**√**		**√**		**√**	**√**	**√**		**√**		**√**	**√**	**√**	**√**	**√**
Health-related quality of life-EuroQol-5D (self-report)	**√**	**√**		**√**		**√**	**√**	**√**		**√**		**√**	**√**	**√**	**√**	**√**
Arrests and incarcerations (criminal record)	Ongoing															
Criminal and Legal Activities Form (self-report)	**√**	**√**		**√**		**√**	**√**	**√**		**√**		**√**	**√**	**√**	**√**	**√**
Opioid use (biochemical)	**√**	**√**	**√**	**√**	**√**	**√**	**√**	**√**	**√**	**√**	**√**	**√**	**√**	**√**	**√**	**√**
Opioid use (self-report)	**√**	**√**	**√**	**√**	**√**	**√**	**√**	**√**	**√**	**√**	**√**	**√**	**√**	**√**	**√**	**√**
Addiction Severity Index	**√**	**√**		**√**		**√**	**√**	**√**		**√**		**√**	**√**	**√**	**√**	**√**

The **DATCAP** is a standardized, customizable tool that can be used to estimate the costs of programs in various settings, and allows for the estimation of both *accounting* and *economic costs* [43, 44]. *Accounting costs* are defined as “the actual expenditures and depreciation of all resources used by the treatment program.” [43] *Economic costs* are considered to be *accounting costs*, plus the value of any resources used by the program that are either subsidized or donated. Including *economic costs* increases generalizability of the estimates, since it is unlikely that resources will be subsidized or donated in all instances.

**Non-study Medical and Other Services (NMOS).** The use of non-study medical services (e.g., inpatient, outpatient, and ED services, and non-study medications) is being assessed using Timeline Follow-Back (TLFB) [45] methodology via the NMOS form. The use of other non-study resources (e.g., workplace productivity, travel time to medical care) will also be self-reported and collected by the NMOS form. The validity of self-reported data on healthcare utilization is well established over recall periods similar to those in our study [46–49]. The NMOS form has been successfully used by our team in prior economic evaluation studies [40, 50–53], and is ideal for capturing the utilization of all relevant non-study resources for this project.

**Health-related quality of life (HRQoL)** is measured by the EuroQol 5D, 3 level (EQ-5D-3L) instrument [54–56]. The EQ-5D is the most widely-used generic, preference-based HRQoL instrument [57]. The EQ-5D-3L measures HRQoL across 5 dimensions: *mobility*, *self*-*care*, *usual activities*, *pain/discomfort*, and *anxiety/depression*, and consists of 3 levels for each domain: *no problems*, *some problems*, and *extreme problems*. The EQ-5D-3L is capable of generating a single health utility index value based on the respondent’s scores for each domain. The health-utility value represents the general US population’s preference for the respondent’s current health state. The health-utility value produced by the EQ-5D-3L can range from − 0.594 to 1, where 0 represents death, 1 represents perfect health, and values below 0 represent health states perceived to be worse than death. The index value can then be used to calculate quality-adjusted life-years (QALYs), as our team, and others have done in similar studies [7, 40, 50, 52].

**Re-arrest and re-incarceration** information will be obtained using official criminal records. These data will include type (e.g., charges involved), number of arrests, convictions, incarcerations, and the length of time of each imposed disciplinary period. We anticipate that most recidivism will be captured by the official records described above; however, we are supplementing these records with self-reported data from the **Criminal and Legal Activities Form (CLAF)** to ensure comprehensive measurement of criminal justice resources utilized, including those relevant to the societal perspective that are not captured in criminal records. The CLAF uses TLFB methodology to capture: days of criminal activity; average number of crimes per day on days when illegal activity occurred; days incarcerated; specific crimes committed; whether the participant was charged with or convicted of the crime; visits to the individual’s parole/probation officer; and parole/probation violations. The self-report method for collecting data on criminal activity has acceptable validity and reliability [58], including among individuals with a substance use disorder, and those on probation or parole [59, 60]. The CLAF has been successfully used by our team in prior studies to value criminal activity and criminal justice resources from various perspectives [40, 50–52], and we recently developed recommendations on how to use different measures of criminal activity in economic evaluations [61].

**Opioid use** is being assessed biochemically via urine or saliva drug tests prior to administration of XR-NTX in prison and the community, and supplemented with self-reported use of opioids (and alcohol and other drugs), measured using the TLFB method.

**Addiction Severity Index (ASI) [**62**] with TLFB**. The ASI is being used to assesses the severity of drug and alcohol use disorders, and the effects of opioid use on the participant’s psychiatric, legal, medical, employment, and family functioning. A composite score can be created for each of these seven areas that provides a valid and reliable measure of the participant’s status. The composite scores will be evaluated for potential use as covariates in our empirical models. We will also utilize the ASI data on substance use frequency and criminal activity to supplement biochemical opioid use and criminal record data.

**Opioid use severity.** We will use the DSM-V opioid-related-disorder classification [63], collected as part of the inclusion/exclusion criteria, to assess opioid use severity (mild, moderate, severe) at baseline. Opioid use severity will be evaluated for potential use as a covariate in our empirical models.

**Unit costs.** Sources of our unit cost estimates are listed in Table 2. The cost of the study-provided therapy will be based on the resources utilized to deliver the 380 mg injection of XR-NTX, which includes the dispensing fee that will be calculated using the DATCAP. For the state policymaker perspective, we will obtain and use the Transformed Medicaid Statistical Information System (T-MSIS) data from the Centers for Medicare and Medicaid Services (CMS), to calculate the cost of all medications and healthcare resources [64]. The T-MSIS data contains the following information from all 50 states, Washington DC, and two US territories (Virgin Islands and Puerto Rico): enhanced information about beneficiary eligibility, beneficiary and provider enrollment service utilization, claims and managed care data, and expenditure data for Medicaid and CHIP. The costs from the T-MSIS database will be calculated for individuals with a DSM-V diagnosed opioid use disorder who are in an age-range reflective of our final study samples.

**Table 2:** Unit Cost Sources for Economic Evaluation

Measure	Utilization data source	Unit cost source–policymaker perspective	Unit cost source–societal perspective
***Substance use disorder therapy***			
XR-NTX	Study documents & NMOS	T-MSIS + dispensing fee estimated via DATCAP	FSS + dispensing fee estimated via DATCAP
Methadone and buprenorphine	NMOS	T-MSIS	FSS
Behavioral therapy	NMOS	T-MSIS	Medicare FFS
Inpatient detoxification	NMOS	T-MSIS	Medicare FFS
Residential treatment	NMOS	T-MSIS	Medicare FFS
***Other healthcare services***			
Hospital stays	NMOS	T-MSIS	Medicare FFS
Outpatient visits	NMOS	T-MSIS	Medicare FFS
Emergency department visits	NMOS	T-MSIS	Medicare FFS
Mental health visits	NMOS	T-MSIS	Medicare FFS
Other prescriptions	NMOS	T-MSIS	FSS
***Criminal justice activities***			
Specific crimes	Criminal records & CLAF	McCollister et al. (2010): direct costs	McCollister et al. (2010): societal costs
Probation visits	CLAF	BLS	BLS
***Other resources***			
Work	NMOS	N/A	Self-reported wages & hours worked
Education	NMOS	N/A	Self-reported time in school & Card (1999) estimates
Participant time	NMOS	N/A	Self-reported or federal minimum wage
Participant travel	Site visit	N/A	Area public transportation fees & IRS mileage rates

*NMOS* Non-study Medical and Other Services form, *T-MSIS* Transformed Medicaid Statistical Information System, *DATCAP* Drug Abuse Treatment Cost Analysis Program instrument, *FSS* Department of Veterans Affairs Federal Supply Schedule, *CLAF* Criminal and Legal Activities Form, *Medicare FFS* Medicare fee-for-service

The societal perspective should account for the “actual value” of all resources associated with the intervention [37]. Thus, Medicare fee-for-service payments will be used to value healthcare resources utilized from the societal perspective, as these payments are designed to reimburse providers for the resources that would be used to treat a typical patient with a given condition and are adjusted for relevant factors that are unique to the patient or provider [65], as opposed to also including a component for profit and risk adjustment [37]. Similarly, the US Department of Veterans Affairs Federal Supply Schedule will be used to value all medications in the societal perspective, as recommended by the Second Panel on Cost Effectiveness in Health and Medicine [37].

Unit cost estimates developed by McCollister et al. [66] will be used to value the resources associated with specific crimes committed. These costs include the direct costs to the criminal justice system (e.g., police-protection, legal and adjudication, and corrections costs), which will be used to inform the state policymaker perspective, as well as societal costs that combine the criminal-justice-system costs with those incurred by victims, both tangible (e.g., medical, property damage or loss, etc.) and intangible (i.e., pain and suffering). Probation officers’ time will be valued according to the mean annual salary and benefit rate reported by the Bureau of Labor Statistics (BLS) [67].

The benefits of work-force participation will be estimated according to the participant’s self-reported wage rate and time spent working. Educational activities will be valued according to self-reported time in school and the estimated return for a year of schooling in the United States [68], applied to the lifetime earnings for individuals in the relevant age range [69]. Participant time costs will be calculated using the self-reported amount of time spent on healthcare-related activities (including travel) and the estimated school or workforce value of the participant’s time [37]. We will use the federal minimum wage to value the time for individuals who are unemployed. If we are able to obtain person-level travel information, including distance traveled and mode of transportation, we will calculate the direct costs of travel accordingly. The cost of public transportation in the area will be used to value the out-of-pocket transportation costs for those using public transit, and IRS standard mileage rates will be used to value transportation costs for those with private transportation. Otherwise, participant transportation costs will be valued according to the average distance traveled by participants, weighted by the proportion who used public transportation versus private transportation, and their respective costs, which will be provided by study staff during site visits.

## Outcomes

The study is comprised of four components, each with its own outcome(s), that culminate in cost-effectiveness analyses. First, we will estimate the cost to the correctional health system of implementing and running each XR-NTX program. Second, we will evaluate differences in the utilization of healthcare services associated with opioid use, across the different arms. Specifically, we will evaluate differences in number of ED, inpatient, primary care, and behavioral healthcare visits. Third, we will evaluate differences in QALYs gained across arms. Finally, we will conduct cost-effectiveness analyses, the primary outcome for which will be the incremental cost-effectiveness ratio (ICER). An ICER is calculated as the incremental cost of a chosen strategy relative to another, divided by the incremental effectiveness of the strategy of interest relative to the other. We will use two measures of effectiveness, one economic, and one clinical. The economic measure of effectiveness will be the QALY. The QALY is an ideal denominator for a cost-effectiveness analysis, as it provides a common measure of effectiveness that permits comparisons across disorders, diseases, and interventions. In addition, generally accepted thresholds for defining value have been established for QALYs, unlike clinical measures [70, 71]. The clinical measure of effectiveness will be *Abstinent Years*, the proportion of the year the individual was abstinent from opioids. Time abstinent is an important measure of effectiveness for clinical stakeholders, and calculating cost-per-*Abstinent*-*Year* enables comparisons with existing economic evaluations that have utilized similar effectiveness measures, especially those that have relied solely on time abstinent measures [7].

## Analysis

The first step of estimating the cost to the correctional health system of implementing and running each XR-NTX program will be to tailor the DATCAP to each setting. Next, we will conduct focused interviews of administrators and staff associated with each treatment strategy across Studies A and B, to ensure that all resources required to implement and continuously manage them, are being captured. The implementation cost will include one-time fixed costs associated with starting the intervention, as well as all monthly fixed and variable costs associated with resources that will be required to manage each treatment strategy on an ongoing basis. Through our interviews, we will estimate the number of patients that could be treated using each strategy on a daily basis, given the current resources, and subsequently calculate the mean costs. The one-time fixed costs associated with starting each strategy will be reported separately; that is, they will not be included in the subsequent economic analyses since they become negligible over time. Research-specific costs will be excluded.

All participant-level data will be analyzed under an intent-to-treat principle, and all analyses will be conducted using a multivariable Generalized Linear Mixed Model (GLMM). The GLMM is an extension of the GLM that allows for the inclusion of random effects. The multivariable aspect of the model is crucial both for the within study comparisons of treatment arms, and the between study comparison of treatment arms, as it allows for the control of factors that are unbalanced between arms because they were not accounted for in the randomization process or may have become unbalanced over time due to loss to follow-up. The GLMM allows one to choose the most appropriate mean and variance functions according to the observed data, and uses all available data for each participant, regardless of whether or not it is complete, making it an ideal statistical procedure for intent-to-treat approaches [38]. Given the differences in mechanisms to generate data, separate multivariable GLMMs will be estimated to predict the mean value for each resource and outcome, at each time period, by study arm. The method of recycled predictions will be used to obtain the final predicted mean values [38]. To account for sampling uncertainty in point estimates, the p-values and standard errors will be estimated using nonparametric bootstrapping techniques within the multivariable framework. All monetary values will be adjusted for inflation.

For Study A (Woody, PI) we will model the person period for each 3-month period where detailed assessments are given to participants (i.e., months 3 and 6). For Study B (Gordon, PI) we will model the person period monthly for the first 7 months and the 5-month follow-up period. By analyzing the relevant person-period for each study, according to when detailed assessments are given to participants, we are able to utilize all observed data from each participant, while accurately identifying and dealing with missing data, and, in addition to the potential confounders discussed above, controlling for differences between arms over time [38.] Next, we will aggregate the monthly values from Study B to align with the 3 and 6 month observation periods of Study B, merge the two datasets, and model the 3- and 6-month person periods.

To better understand the differences in healthcare resource utilization across treatment strategies, we will test for differences in the predicted number of ED, inpatient, primary care, and behavioral healthcare visits between study arms both within- and across-studies over the course of the intervention and follow-up periods, using the methods described above. Similarly, to estimate whether health-related quality-of-life varies across treatment strategies, the predicted health utility index values obtained from the GLMM will be used to estimate QALYs gained using the area under the curve methodology [37, 38, 72], which will then be tested in a similar fashion.

Healthcare services utilized will then be weighted by their respective unit costs and predicted values will be generated. The predicted mean costs for each resource category will be summed and tested according to the relevant perspective, and incorporated into a comprehensive cost-effectiveness analysis along with the predicted outcomes and the average ongoing management costs of each strategy. Four ICERs will be constructed for Study A (Woody, PI), Study B (Gordon, PI), and the combined analysis; i.e., an ICER for each effectiveness measure (QALYs and *Abstinent Years*) and each perspective (state-policymaker and societal). ICER confidence intervals will be estimated using nonparametric bootstrapping techniques within the multivariable framework. Parametric methods based on parameters obtained from bootstrapping will be used to estimate acceptability curves, which illustrate the probability that the intervention is a good value for different willingness-to-pay thresholds (i.e., cost-per-QALY and cost-per-*Abstinent*-*Year*). ICERs will be calculated and acceptability curves will be constructed regardless of the statistical significance for individual cost and effectiveness differences, as the power to detect a difference in costs and effects jointly exceeds the power to do so individually [38].

Sensitivity analyses will be performed to account for uncertainty in assumptions and parameter estimates applied in the analysis [37]. For example, values estimated using the relatively robust and efficient GLMM regression will be compared to those estimated using the more transparent ordinary least squares regression, as well as to the unadjusted mean values. We will also vary the prices of resources, especially the price of the XR-NTX injection, as our prior analyses revealed sensitivity in the cost-effectiveness outcomes around the XR-NTX price [40, 51].

## Timeline

This is a 4 year study, designed to coincide with the timelines of Study A (Woody, PI) and B (Gordon, PI); we are currently in the beginning of Year 2. The first step of the analysis plan, estimating the cost to the correctional health system of implementing and running each XR-NTX program, is in process for both studies. Study A was recently completed and is in the process of conducting analyses of their primary outcomes; thus, we will be receiving the trial data soon to begin the within-study analyses of healthcare resource utilization, QALYs, and cost-effectiveness. Study B (Gordon, PI) is in Year 3 of 5 and is nearing completion of recruitment/randomization. We anticipate that the follow-up assessments for Study B will be complete late in Year 3 of this project.

## Discussion

A notable treatment gap persists among the high-risk population of justice-involved persons with opioid use disorder. Persons just released from incarceration are at a particularly high risk for opioid overdose and overdose deaths. Evidence suggests that evidence-based pharmacotherapy just prior to release is associated with positive outcomes; unfortunately, barriers to access of these opioid use disorder treatments exist for this population. XR-NTX has some characteristics that make it relatively attractive to many prison/jail systems, and it provides protection from opioid overdose for approximately 30 days; however, the high cost of the medication represents yet another barrier to access. It is critical that the policymakers who are ultimately setting prison/jail healthcare budgets [34], and making decisions on behalf of taxpayers and society as a whole, do not view the cost of opioid use disorder therapy for this high-risk population, in isolation. This protocol describes an innovative study that will fill a critical knowledge-gap in the literature and inform the “real-world” resource allocation decisions faced by policymakers and correctional health systems. We will analyze data from two randomized clinical trials, independently and together, to evaluate whether XR-NTX treatment is associated with expected changes in the utilization of healthcare services, enhanced patient wellbeing, and economic viability from state policymaker and societal perspectives among the previously-unstudied, high-risk population of persons with opioid use disorder who are being released from incarceration. We will also have the unprecedented opportunity to measure health-related quality-of-life (HRQoL) among persons with opioid use disorder who were incarcerated, prior to their release, and then track changes in their HRQoL following their release and subsequent engagement with treatment.

## Limitations

The generalizability of our findings may be limited to justice-involved persons with an opioid use disorder; however, we believe this study is highly significant due to (a) the size of the US criminal justice population, (b) the rate of drug use disorders among this population, and (c) the concomitant personal and public health costs. The study relies heavily on self-reported data; however, the validity of self-reported data on healthcare use is well established over recall periods, and among populations, similar to those in our study, and our team has successfully completed numerous economic evaluations using the same instruments proposed here. Although EHR or administrative claims datasets are likely to capture a more detailed set of services than self-report forms, these data are generally limited in their ability to capture and value all relevant healthcare resource utilization among trial participants. The study sample is likely to consist of individuals who lack health insurance; for example, in our prior study on community-dwelling, criminal-justice-involved persons with an opioid use disorder, 29% of participants were uninsured [40, 41]. The data for those who are insured would likely require access to a number of different administrative systems, which would not be feasible. Even if we could access all systems, only a limited subsample may agree to release their health insurance records [73]. Also, the use of claims data limits the evaluation to those healthcare items that are covered, and periods when the individual was covered; moreover, the monetary values in these data often reflect reported charges, as opposed to costs, and the conversion of charges to costs can be inaccurate [74].

## Conclusion

The opioid epidemic continues to escalate, and persons being released from incarceration are at especially high risk for opioid relapse, overdose, and overdose death immediately following release. Furthermore, this population faces a number of significant barriers to obtaining evidence-based therapy. Currently, XR-NTX has the highest rate of acceptance in criminal justice settings [75], yet its relatively high cost serves as a potential barrier to access if viewed myopically. This study is uniquely positioned to deepen our understanding of the impact that XR-NTX treatment for opioid use disorder could have in the criminal justice system, the spillover effects to society as a whole, and the economic viability of alternative delivery strategies, according to different stakeholder perspectives.

## Data Availability

Not Applicable.

## Abbreviations

XR-NTX: Extended-release naltrexone
NIDA: National Institute of Drug Abuse
ED: Emergency department
DATCAP: Drug Abuse Treatment Cost Analysis Program
T-MSIS: Transformed Medicaid Statistical Information System
NMOS: Non-study Medical and Other Services
TLFB: Timeline follow back
HRQoL: Health-related quality-of-life
QALY: Quality adjusted life year
CLAF: Criminal and Legal Activities Form
ASI: Addiction Severity Index
DSM-5: Diagnostic and Statistical Manual of Mental Disorders
BLS: Bureau of Labor Statistics
ICER: Incremental cost effectiveness ratio
GLMM: Generalized linear mixed models
EHR: Electronic health record
